# Overcompensation: a 30‐year perspective

**DOI:** 10.1002/ecy.2667

**Published:** 2019-03-26

**Authors:** Satu Ramula, Ken N. Paige, Tommy Lennartsson, Juha Tuomi

**Affiliations:** ^1^ Department of Biology University of Turku Turku 20014 Finland; ^2^ School of Integrative Biology University of Illinois at Urbana‐Champaign 505 South Goodwin Avenue Urbana Illinois 61801 USA; ^3^ Swedish Biodiversity Centre Swedish University of Agricultural Sciences Box 7016 Uppsala 75007 Sweden

**Keywords:** herbivory, overcompensation, overcompensation 30 years later, plant defense, plant–herbivore interactions, resistance, tolerance

## Abstract

Biomass removal by herbivores usually incurs a fitness cost for the attacked plants, with the total cost per unit lost tissue depending on the value of the removed tissue (i.e., how costly it is to be replaced by regrowth). Optimal defense theory, first outlined in the 1960s and 1970s, predicted that these fitness costs result in an arms race between plants and herbivores, in which selection favors resistance strategies that either repel herbivores through morphological and chemical resistance traits in order to reduce their consumption, or result in enemy escape through rapid growth or by timing the growth or flowering to the periods when herbivores are absent. Such resistance against herbivores would most likely evolve when herbivores are abundant, cause extensive damage, and consume valuable plant tissues. The purpose of this Special Feature is to celebrate the 30th anniversary of the phenomenon of overcompensation, specifically, where the finding has brought us and where it is leading us 30 yr later. We first provide a short overview of how the phenomenon of overcompensation has led to broader studies on plant tolerance to herbivory, summarize key findings, and then discuss some promising new directions in light of six featured research papers.

## Tolerance and Overcompensation

**Figure 1:**
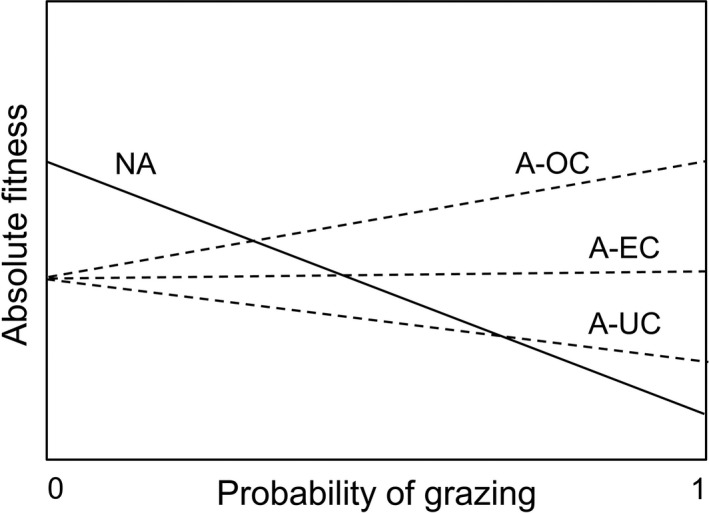
Absolute fitness of nonadapted (NA) and adapted (A) plants in relation to the probability of grazing, following Järemo et al. (1999). Three possible responses to grazing are shown for adapted plants: their absolute fitness can either decline compared to undamaged, adapted plants (undercompensation, A‐UC), remain unaffected (equal compensation, A‐EC), or increase above the level of undamaged, adapted plants (overcompensation, A‐OC). Note that nonadapted plants suffer from an increasing cost of herbivory with increasing grazing, whereas adapted plants pay the price for their adaptive (tolerance) traits in the absence of grazing.

Although numerous observational and experimental ecological studies have demonstrated the costs of herbivory on plant growth or reproduction, costs do not appear to be a universal rule. For example, moderate mammalian grazing increased net biomass production in Serengeti grasses and sedges (McNaughton 1979, 1983). A debate over the potential advantages of grazing for plants began in the 1980s and was further fueled by the intriguing observation that in an Arizona population of the biennial herb scarlet gilia (*Ipomopsis aggregata*), browsed plants branched vigorously and produced a greater number of fruits and viable seeds than unbranched, intact plants (Paige and Whitham 1987). The latter observation was particularly striking because it demonstrated overcompensation to be an unambiguous fitness parameter in a monocarpic plant. This finding led to an important conceptual refinement of “plant defenses” to include tolerance traits (Belsky et al. 1993, Rosenthal and Kotanen 1994, Strauss and Agrawal 1999, Juenger and Lennartsson 2000, Stowe et al. 2000) that mitigate the potentially negative effects of herbivore damage on plant performance and lifetime reproductive success, placing regrowth strategies on par with chemical defense strategies. Moreover, the finding inspired conceptual theories about the mechanisms for overcompensation (Whitham et al. 1991, Fornoni 2011) and the conditions under which such mechanisms are likely to evolve (Crawley 1987, Maschinski and Whitham 1989, Paige 1992, Wise and Abrahamson 2005, 2008). It has also been shown that the degree of tolerance in plants ranges from undercompensation to complete compensation, or even overcompensation (Fig. 1; Maschinski and Whitham 1989; see also Belsky 1986 for a subdivision), with a potential (over)compensation peak occurring at either low, moderate, or high damage levels (McNaughton 1983, Paige 1994, Tuomi et al. 1994, Nilsson et al. 1996, Huhta et al. 2003).

## Genetic Basis of Tolerance

Recent studies in few herbaceous species have uncovered several key molecular mechanisms involved in the compensatory continuum. For example, a combinatorial approach using quantitative trait locus mapping and microarray analysis uncovered a gene, glucose‐6‐phosphate‐1‐dehydrogenase (G6PD1, At5 g35790.1), that plays a major role in controlling tolerance in *Arabidopsis thaliana*. This gene is the central regulatory enzyme in plant metabolism that generates nicotinamide adenine dinucleotide phosphate and a variety of metabolic intermediates for biosynthetic processes (Siddappaji et al. 2013). Knockout studies and complementation studies verify G6PD1's role in the tolerance response. Three different T‐DNA insertion lines used in a G6PD1 knockout study in the *Arabidopsis* Columbia‐4 background displayed patterns of equal compensation, with a trend toward undercompensation, in contrast to overcompensation, as observed in wild‐type Columbia following experimental clipping. Gene complementation of G6PD1 in the T‐DNA line also restored the compensatory response from equal compensation to overcompensation as exhibited in wild‐type Columbia. Moreover, in the annual herb *Nicotiana attenuata*, SNF1‐related protein kinases have been demonstrated to alter plant resource allocation and, consequently, improve tolerance to herbivory (Schwachtje et al. 2006).

The ability of a plant to increase its ploidy level via endoreduplication (which occurs in the majority of herbaceous plants) leads to rapid regrowth and an increase in fitness following the removal of apical dominance explaining, in part, plant tolerance (Scholes and Paige 2014). Endopolyploidy is the presence of several ploidy levels within the same plant, resulting from the process of endoreduplication, where the cell forgoes mitosis, increasing cellular ploidy. Growth by cell division and expansion through endoreduplication may provide quicker growth than by cell division alone (Barow 2006). In addition, increasing chromosome number through endoreduplication and therefore gene copy number may provide a means of increasing gene expression of vital gene pathways that promote rapid regrowth rates following herbivory. High levels of auxin are known to repress the endocycle, whereas lower levels of auxin trigger an entry into the endocycle and an exit from mitotic cycles (Ishida et al. 2010). Importantly, removal of apical dominance leads to a reduction in the level of auxin leading to axillary bud break and consequent stem regeneration. Therefore, there is a direct link between the endocycle and the removal of apical dominance (a frequent form of natural herbivory in *A. thaliana*, Weinig et al. 2003). Both a positive correlation between tolerance and endoreduplication and a causal link between endoreduplication and the degree of fitness compensation have been established (Scholes and Paige 2011, 2014).

## Tolerance and Resistance

In the arms race framework, overcompensation can be treated as an expression of plant tolerance that may be selected for when herbivore damage is predictable and extensive (e.g., Crawley 1987, Paige and Whitham 1987, Vail 1992, Tuomi et al. 1994, Lennartsson et al. 1997). From an evolutionary perspective, it was initially theorized that resistance and tolerance represented two alternative, redundant, defense strategies (or resistance strategies sensu Belsky et al. 1993) that would lead to a trade‐off between them; that is, high constitutive resistance would be expected to co‐occur with poor tolerance and vice versa (van der Meijden et al. 1988, Vail 1992, Mauricio et al. 1997). According to this hypothesis, both defensive strategies offer the same fitness benefits (van der Meijden et al. 1988, Mauricio et al. 1997). A trade‐off may be due to resource allocation, but it may also occur for evolutionary reasons. If the evolution of overcompensation requires a predictable, high risk of damage (Crawley 1987), then strategies for reducing the damage rate should not be selected for in overcompensating plants. Regardless of the suggestions that resistance and tolerance may play redundant roles in plant defense, a previous meta‐analysis showed most natural populations appear to be comprised of a mixture of resistance–tolerance strategies because of selection for the maintenance of both traits (Leimu and Koricheva 2006).

The diversity of resistance–tolerance relationships, confirmed by the findings above, may be explained by the herbivore community as a selective force for plant defense strategies. For example, a trade‐off traditionally assumed between resistance and tolerance may occur under certain conditions only (e.g., in the presence of a few specialist herbivores), whereas high herbivore diversity can be assumed to select for mixed resistance–tolerance strategies in most cases (Turley et al. 2013). It is also possible that mixed strategies evolve because of positive relationships between resistance and tolerance. As an example, tolerance mechanisms may not provide sufficient compensatory growth if the plant is grazed several times within a growing season (Tuomi et al. 1994, Nilsson et al. 1996, Stowe et al. 2000), and therefore, both resistance and tolerance mechanisms are selected for simultaneously, given that they reduce the rate of repeated damage. In summary, more studies are needed in order to reveal which environmental conditions and evolutionary mechanisms explain the continuum of plant responses from mixed to single strategies.

## Tolerance is Influenced by Abiotic and Biotic Factors

The ecological context is known to affect the magnitude and even the direction of plant tolerance (Abrams 1987, Chamberlain et al. 2014, Garcia and Eubanks 2019, Pearse et al. 2017, Poveda et al. 2018, this Special Feature). As an example, the indirect positive effects of herbivores on plant performance are mediated by improved soil nutrient pools or by the removal of competitors (de Mazancourt et al. 2001). Although high tolerance is generally expected in nutrient‐rich environments with low levels of competition (Maschinski and Whitham 1989; but see Hawkes and Sullivan 2001, Rautio et al. 2005, Allsup and Paige 2016), plant responses to herbivory are likely to vary, depending on the resources that are limiting plant (re)growth (Wise and Abrahamson 2007). For example, in seasonal environments, periods of drought can hinder overcompensation (Lennartsson et al. 1998, Levine and Paige 2004). Observations of overcompensation in the field and in manipulative experiments have demonstrated that context dependence also applies to the direct effects of herbivore consumption on plant fitness. Such direct effects have sometimes been suggested to imply plant–herbivore mutualism (Vail 1992, Agrawal 2000). However, it should be noted that so far the context dependence of overcompensation in plants is restricted to the timing of damage in relation to flowering time, the degree and frequency of shoot damage, plant size, resource availability, and soil quality.

When analyzing context dependence of plant defense strategies and, in particular, when discussing plant–herbivore mutualism and “the advantages of being eaten”, we may need to conceptually separate current adaptive advantages from evolutionary advantages (e.g., see Agrawal 2000). Overcompensating plant species may simply reflect a current advantage of being eaten because they have evolved tolerance mechanisms that require grazing to maximize fitness (de Mazancourt et al. 2005). However, overcompensation does not necessarily indicate that plant performance in the presence of herbivores has increased compared to “ancestral” plants in the absence of herbivores (Mathews 1994, Tuomi et al. 1994, Järemo et al. 1999; see evolutionary dependence in de Mazancourt et al. 2005). From such an ecosystem perspective, the evolutionary advantages of herbivory can be seen as highly hypothetical (McNaughton 1986, Crawley 1987). Overall, to understand diverse plant defense strategies better, it is essential to explicitly link them to the ecological context, as illustrated by several papers included in this Special Feature.

## New Avenues for Understanding Tolerance

This Special Feature sheds light on plant tolerance to herbivory through six research papers that cover topics from the mechanistic understanding of tolerance and resistance to the potential application of overcompensation in agriculture. Mesa et al. (2017; this Special Feature) demonstrate in *A. thaliana* that assumed trade‐offs between resistance and tolerance are unlikely, given that resistance and tolerance are evolutionarily constrained by co‐localization in the oxidative pentose phosphate pathway and the joint expression of resistance and tolerance dictated by the degree of endoreduplication. This finding emphasizes the importance of considering molecular constraints in studies on plant defense strategies. So far, evidence of molecular mechanisms for tolerance primarily comes from a couple of herbaceous species (*A. thaliana* and its ecotypes, and *N. attenuata*), and future studies should therefore consider a wider range of plants that represent both herbaceous and woody species. The genetic background of overcompensation is lacking for most of the species in which overcompensation has been demonstrated, which may partially limit the chances of revealing the evolutionary background of overcompensation. We clearly need more empirical data on the genetic basis of tolerance and resistance, not merely for investigating genetic correlations between the two, but also for evaluating model assumptions and for linking tolerance to plant architectural potential and constraints.

To date, nearly all comparisons of tolerance among species rely on meta‐analysis of tolerance studies and comparisons between plants from disparate plant families (but see Agrawal and Fishbein 2008). In this Special Feature, Pearse et al. (2017) estimated tolerance to folivory, tolerance to “browsing” (through clipping), and resistance to two lepidopteran herbivores for a number of species in the *Streptanthus* clade. Using phylogenetically informed analyses, they found that tolerance was not constrained by phylogeny. Moreover, tolerance to folivores was unrelated to the tolerance of a plant species to browsing, but was associated with late flowering times, suggesting that plants that invest early in reproduction do so at a cost to tolerance. No evidence was found for a resistance–tolerance trade‐off in the *Streptanthus* clade, indicating that tolerance is constrained by other factors than resistance. Such phylogenetically controlled comparisons of taxa and populations are an interesting potential avenue for discovering physiological mechanisms and adaptive characters behind the evolution of tolerant lineages. Moreover, this approach can reveal geographical patterns of tolerance that can be then compared with historical environmental conditions and the selective pressures of other herbivores within the community.

Recent studies have shown that the capacity of plants to tolerate damage partially depends on their predamage growth patterns (Strauss and Agrawal 1999, Hochwender et al. 2000, Scholes et al. 2017). However, the majority of these studies have focused on adult stages in native species, ignoring the fact that mechanisms may differ across life stages (Barton 2013) and between invasive and noninvasive species (Ashton and Lerdau 2008). Regardless of the evolution of increased competitive ability (EICA) hypothesis (Blossey and Nötzold 1995) stating that invasive species have improved their competitive ability because of changes in resource allocation as a response to lack of native herbivores, surprisingly little is known about tolerance relative to species invasiveness. In this Special Feature, Lurie et al. (2017) investigate plant traits associated with the seedling tolerance of invasive and noninvasive tree species in Hawaii, and demonstrate that predamage growth patterns determine tolerance independent of species’ invasiveness status. Their finding supports the view that the same tolerance mechanism may act early in life for both invasive and noninvasive woody species, and might be generalizable to some extent across species. No doubt, comparison of populations in their native and introduced ranges that differ in herbivory pressure represents another interesting avenue for studying the costs and benefits of adaptations to herbivory.

Compensatory production of new branches can occur if the plant has dormant meristems that are triggered to grow by damage. Meristem suppression is usually caused by apical dominance, and it has also been proposed that overcompensation may be a by‐product of selection for plant tallness in response to intense competition for light or pollinators (Aarssen and Irwin 1991, Aarssen 1995). Lennartsson et al. (2018; this Special Feature) use a field experiment combined with structured population models in two subspecies of *Gentianella*, to test whether restrained branching caused by apical dominance creates a competitive advantage in tall vegetation and/or an increased capacity for compensatory growth after damage. Based on long‐term population growth rates under different gradients of competition and clipping damage, they show that restrained branching in *Gentianella* cannot be selected for by competition alone, but that episodes of apical damage are required to maintain the trait. The findings by Lennartsson et al. (2018), on the other hand, suggest that selection for tolerance may be involved in the evolution of traits that are not usually connected to plant defense strategies; for example, apical dominance may act as a mechanism for tolerance in frequently grazed habitats. Moreover, the study by Lennartsson et al. (2018) highlights that structured population models considering the entire life cycle of the species (Caswell 2001) provide a useful tool for translating individual plant responses to herbivory into a population level. Population‐level data are also needed in a conservation context, where it is important to match conservation activities and land‐use regimes with plant adaptations and to evaluate the effect of management actions in terms of population viability.

Overcompensation can be economically important through its potential applications in agriculture. Poveda and her colleagues have previously shown that low level herbivory by the Guatemalan tuber moth can lead to a doubling in yield of a Columbian potato variety (e.g., Poveda et al. 2018). Because compensatory responses appear to be context‐dependent, in this Special Feature, Poveda et al. ( 2018) assess whether changes in the biotic and abiotic environment, such as those that occur along altitudinal or landscape gradients, may influence the plant compensatory ability in response to herbivore damage. Overall, their results indicate that potato plants maximize productivity when about 10% tubers are damaged, although compensatory responses partially depend on landscape structure, with the highest compensatory responses occurring in simple landscapes that mostly consist of agricultural area within a certain altitudinal range. These results are promising in terms of the use of overcompensation as a mechanism to increase crop productivity in this Columbian potato variety. The same seems plausible also for a wider range of crop plants. For example, a meta‐analysis of insect herbivory across plant growth forms by Garcia and Eubanks (2019, this Special Feature) demonstrates that many other cultivated plants show overcompensatory responses to damage that can be even greater than those of wild, uncultivated plants. As the next step, these findings all together highlight the need for applied research that aims to produce more concrete general guidelines for farmers on how to feasibly increase their yields.

Although a quantitative meta‐analysis provides a way to synthetize accumulated individual studies and to produce generalizations about plant tolerance to herbivory (Garcia and Eubanks 2019, this Special Feature), we are still lacking broad, comparative studies that investigate plant defense strategies in relation to different abiotic and biotic factors and to different genetic and physiological potential and constraints. During the past 30 yr of tolerance and overcompensation studies, there has been a shift from describing patterns to exploring mechanisms, resulting in the rejection of some traditional hypotheses (e.g., a resistance–tolerance trade‐off). This research trend toward a deeper mechanistic understanding of tolerance will continue with the help of recent developments in genetics, phylogenetics, and community and population models. Many of these research directions are treated in this Special Feature, which we hope will further fuel tolerance and overcompensation studies.
